# Meiofauna Metabolism in Suboxic Sediments: Currently Overestimated

**DOI:** 10.1371/journal.pone.0059289

**Published:** 2013-03-28

**Authors:** Ulrike Braeckman, Jan Vanaverbeke, Magda Vincx, Dick van Oevelen, Karline Soetaert

**Affiliations:** 1 Ghent University, Department of Biology, Marine Biology Research Group, Ghent, Belgium; 2 Royal Netherlands Institute for Sea Research - NIOZ-Yerseke, Yerseke, The Netherlands; Universidade Federal do Rio de Janeiro, Brazil

## Abstract

Oxygen is recognized as a structuring factor of metazoan communities in marine sediments. The importance of oxygen as a controlling factor on meiofauna (32 µm-1 mm in size) respiration rates is however less clear. Typically, respiration rates are measured under oxic conditions, after which these rates are used in food web studies to quantify the role of meiofauna in sediment carbon turnover. Sediment oxygen concentration ([O_2_]) is generally far from saturated, implying that (1) current estimates of the role of meiofauna in carbon cycling may be biased and (2) meiofaunal organisms need strategies to survive in oxygen-stressed environments. Two main survival strategies are often hypothesized: 1) frequent migration to oxic layers and 2) morphological adaptation. To evaluate these hypotheses, we (1) used a model of oxygen turnover in the meiofauna body as a function of ambient [O_2_], and (2) performed respiration measurements at a range of [O_2_] conditions. The oxygen turnover model predicts a tight coupling between ambient [O_2_] and meiofauna body [O_2_] with oxygen within the body being consumed in seconds. This fast turnover favors long and slender organisms in sediments with low ambient [O_2_] but even then frequent migration between suboxic and oxic layers is for most organisms not a viable strategy to alleviate oxygen limitation. Respiration rates of all measured meiofauna organisms slowed down in response to decreasing ambient [O_2_], with Nematoda displaying the highest metabolic sensitivity for declining [O_2_] followed by Foraminifera and juvenile Gastropoda. Ostracoda showed a behavioral stress response when ambient [O_2_] reached a critical level. Reduced respiration at low ambient [O_2_] implies that meiofauna in natural, i.e. suboxic, sediments must have a lower metabolism than inferred from earlier respiration rates conducted under oxic conditions. The implications of these findings are discussed for the contribution of meiofauna to carbon cycling in marine sediments.

## Introduction

Oxygen is essential to life and recognized as a structuring factor of metazoan communities in marine sediments [1]. The importance of oxygen as a controlling factor of the respiratory activity of meiofauna (32 µm-1 mm in size) is however less clear. Typically, respiration rates are measured under oxic conditions, after which these respiration rates are used in food web studies to quantify their role in sediment carbon turnover and compare their contribution to other biotic compartments e.g. [2]–[4]. The oxygen concentration ([O_2_]) in sediments is however generally far from fully saturated, implying that current estimates of the role of meiofauna in carbon cycling may be biased.

The availability of oxygen in marine sediments depends on the oxygen demand for organic matter degradation and on supply through several transport mechanisms [1]. In sediments with high organic matter degradation such as muddy coastal sediments, oxygen is generally limited to the surficial millimeters, whereas much lower organic matter degradation in deep-sea sediments renders the sediment column oxygenated for >20 cm [5]. Advection through hydrodynamic forces in permeable sands [6] and irrigation of macrofaunal burrows in muddy sediments [7] are transport mechanisms that may enhance oxygen penetration.

Since nearly all marine animals depend on oxygen [8], most benthic organisms are restricted to the oxygenated sediment layer of the sea floor. Macrofauna is capable of dwelling deeper suboxic layers, because they adopted strategies to stay in contact with the oxygenated overlying water, such as the development of burrows, tubes and siphons [7]. Meiofauna are generally found in oxygenated habitats near the surface or around irrigated macrofaunal burrows (e.g. [3], [9]). Only meiobenthic organisms adapted to low oxygen and high sulphide concentrations can thrive temporally in the suboxic to anoxic layers, for which two main hypotheses are proposed: 1) frequent migration to oxic layers [10]–[13] and 2) adaptation in shape [14]. To evaluate these hypotheses quantitatively, one needs to understand the turnover of oxygen in the meiofaunal body. Oxygen turnover depends on the organism’s ability to take up oxygen from the environment at low oxygen conditions, their metabolic demands and the oxygen storage in the body. Although knowledge on these individual aspects is available, they have not been integrated to quantitatively evaluate oxygen turnover in function of the hypotheses raised above. This is an important issue, since the turnover determines the strength of the coupling between ambient sedimentary [O_2_] on an organism’s metabolism.

To address this question we utilize a mathematical model on oxygen distribution in an organism [15], [16] with which we quantitatively establish the turnover of oxygen in meiobenthos. Since meiobenthos consists mainly of nematodes (>50% in abundance of all habitats globally [17]), we focused on nematodes as an example (but see [15] for a similar analysis on Turbellaria). Secondly, respiration measurements on meiofauna (e.g. [18]–[23], [26] and references therein) have always been conducted at oxic conditions, which are not necessarily realistic field conditions. Early measurements already hinted towards lower metabolism and lower respiration rates for organisms living in suboxic sediments e.g. [15], [24], [25]. Therefore, respiration estimates under realistic environmental conditions (i.e. oxygen deficiency) are necessary. We determined respiration rates on a gradient of [O_2_] ranging from fully oxic to near anoxic for four species belonging to different meiobenthic taxa (Nematoda, Foraminifera, Ostracoda and juvenile Gastropoda).

We find that frequent migration to oxic layers is generally not a viable strategy to alleviate low ambient [O_2_] and that meiofaunal respiration slows down under suboxic conditions. The implications of these findings are discussed for the contribution of meiofauna to carbon cycling in marine sediments.

## Results and Discussion

### Development of Oxygen Turnover Model in a Nematode Body

The body of a nematode can be considered as a cylindrical shape, whereby oxygen uptake occurs through the (body) wall of the cylinder by molecular diffusion. A model of the oxygen budget in a cylinder at steady-state has been described in [15], [16] and is given by:
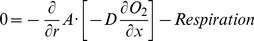



The first term of the equation is transport of oxygen by molecular diffusion, where *A* is the outer surface of the cylinder, *r* is the radial distance, *D* is the diffusion constant. Oxygen is consumed in respiration (*Respiration*) and is considered here a fixed value of 0.5 mol O_2_ l^−1^ d^−1^ [11]–[16], [24] and references in these works.

**Figure 1:**
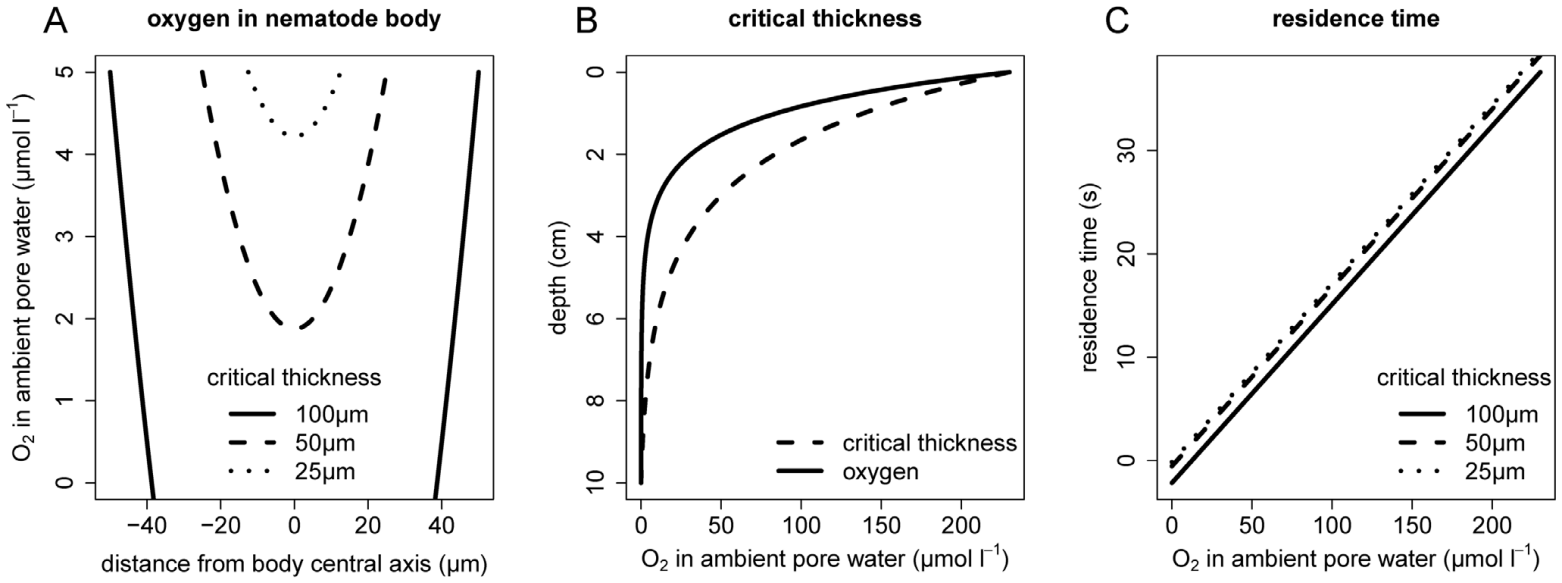
Results of the oxygen turnover model. Oxygen concentration in ambient pore water and body wall as a function of nematode body radius in suboxic conditions (A), nematode critical thickness (µm) as a function of sediment depth (B) and oxygen residence time in nematode body as a function of [O_2_] in ambient pore water from oxic to anoxic conditions (C).

Oxygen enters the body through the wall and is consumed at equal rate in the body. This results in an oxygen gradient in the body with highest concentration at the wall and lowest concentration in the center of the body. The gradient of oxygen as a function of radial distance *r* can be described as follows:
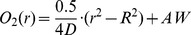



With *r* the distance from the nematode body central axis, *R* the radius of the whole body and *AW* the [O_2_] in the ambient pore water. It appears that [O_2_] inside the body wall depends on the thickness of the nematode [14], [15]. One can define a “critical thickness” or the maximum width of a nematode as a function of the AW [O_2_] after which anoxia inside the body develops (Fig. 1A). Thinner nematodes become anoxic at lower AW [O_2_]. Based on these results, one predicts that nematodes living in less oxygenated environments are long and slender nematodes.

As the oxygen concentration declines with sediment depth, the critical thickness decreases with sediment too. Fig. 1B depicts a typical vertical oxygen profile within a sediment with diffusive oxygen uptake where [O_2_] decreases with depth [1]. The model in Fig. 1B predicts indeed a logarithmic decline of body width (critical thickness) with sediment depth, which confirms the hypothesis put forward by [14] and [15]. In the deeper, suboxic sediment layers, longer nematodes can deal more easily with low [O_2_] than thick nematodes.

These steady-state considerations however do not quantify the residence time of oxygen in a nematode’s body. Fig. 1C depicts the residence time of oxygen in a nematode’s body as a function of the [O_2_] in the ambient pore water in the condition that the oxygen that entered the body through the wall is consumed equally fast in the body. Oxygen storage inside the body may increase the turnover times but the lack of oxygen-binding pigments in most nematodes [27] make storage very unlikely. In all, the residence time of oxygen in the nematode’s body ranges from 1 to 30 s depending on sediment type and oxygen availability. This very fast turnover together with limited nematode moving speed (0.03–3 mm s^−1^ [28], [29], with long and large nematodes moving faster than small nematodes) implies that migration between oxic and suboxic sediment layers is not a viable option, at least not for small nematodes. Moreover, the oxygen condition in the environment directly controls the oxygen availability inside the nematode body and hence directly controls the respiration rate of the nematode. This oxygen dependency is probably a fact for meiofauna in general [15], [16]. Hence, it is crucial to determine the relation between respiration rates of different meiofauna taxa and [O_2_] in the surrounding water, which will be discussed next.

### Meiofaunal Respiration in an Oxic to Anoxic Gradient

**Figure 2:**
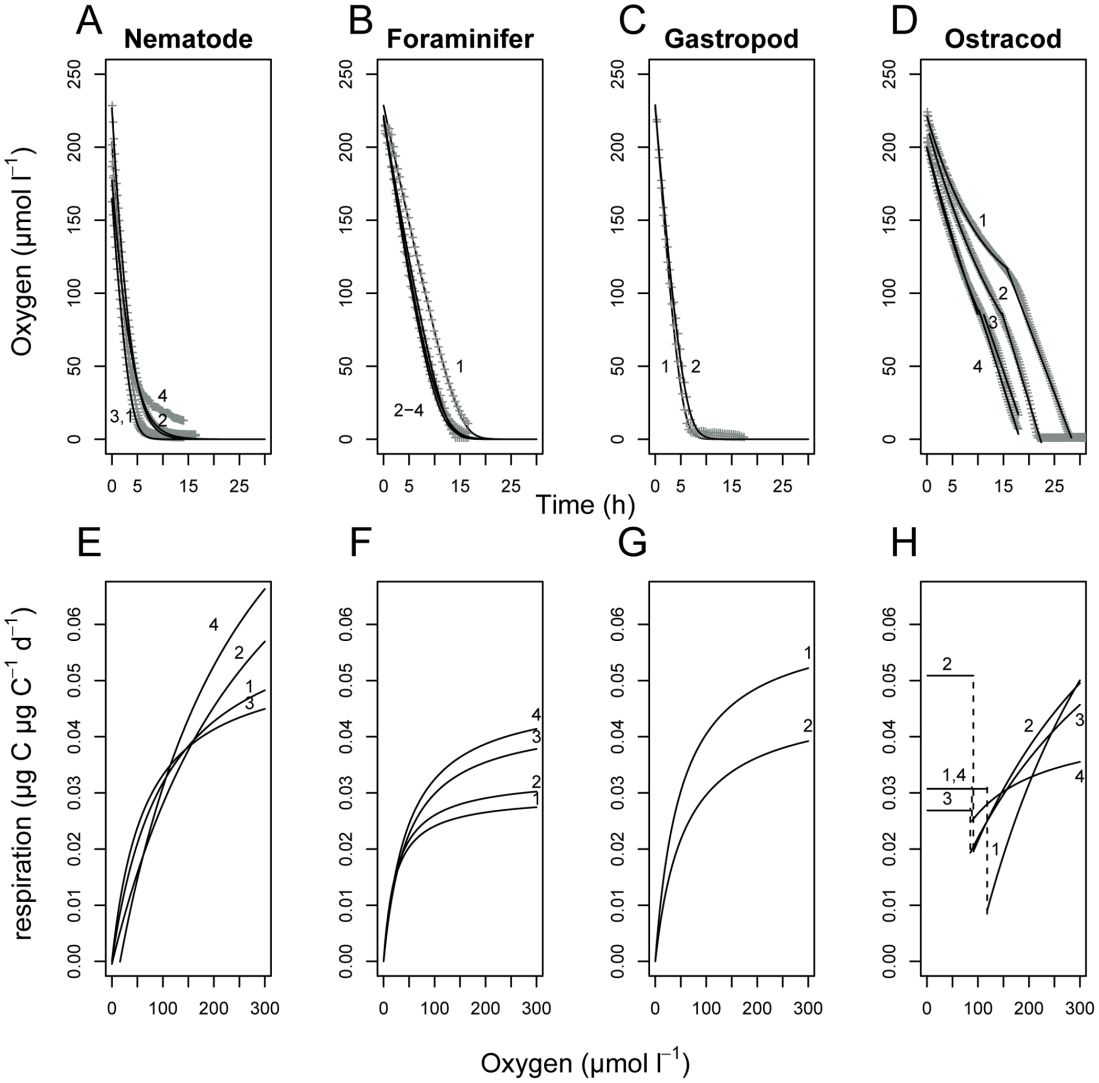
Measured and modeled meiofauna respiration. Observed (grey +) and modeled (line) oxygen consumption of the Nematode *Enoploides longispiculosus*, the Foraminifer *Ammonia beccarii*, the juvenile Gastropod *Hydrobia ulvae* and the undefined Ostracod species (A–D) and corresponding modeled respiration rates as a function of the available oxygen from oxic to anoxic conditions (E–H). Replicate numbers are indicated. The Ostracod model is composed of a Monod and a linear fit. The dashed line in (H) indicates the transition from Monod to linear fit (i.e. stress reaction).

To understand the respiration of meiofaunal organisms as a function of oxygen availability, it is crucial to perform respiration experiments at realistic [O_2_], i.e. going from oxic to suboxic conditions. For oxic conditions [230 µmol O_2_ l^−1^], the respiration rates (R_max__day (100%), Table 1) have already been reported in [30] and fall within the range reported for meiofauna at similar temperature [18]–[23]. During the incubation, [O_2_] slowly drops in the micro-chambers due to respiration. The incubations lasted 5–30 h during which anoxia developed in the chambers. Respiration in all measured meiofauna organisms slowed down as a result of declining [O_2_], however clear taxon-specific differences in sensitivity were observed (Fig. 2). The nematode *Enoploides longispiculosus* henceforth referred to as (“Nematode”) (Fig. 2A), the foraminifer *Ammonia beccarii* (“Foraminifer”) (Fig. 2B) and juvenile gastropod *Hydrobia ulvae* (“Gastropod”) (Fig. 2C) consumed all oxygen in ∼15 hours, but the initial decline of [O_2_] was stronger in the Nematode. Finally, the oxygen decline in the undefined Ostracoda species (“Ostracod”) (Fig. 2D) treatments followed a somewhat different pattern, leveling off at about 50% of oxygen saturation, but after which a steep decrease in [O_2_] to zero oxygen occurred at 17–30 h.

**Table 1:** Coefficients of the non-linear models for the respiration measurements of the Foraminifer *Ammonia beccarii*, the Nematode *Enoploides longispiculosus* and the juvenile Gastropod *Hydrobia ulvae*.

Treatment	k_s_	O_2 initial_	O_2,min_	Biomass	R_max__day (100%)	R_max__day (10%)	R_max__day (1%)
	(µmol O_2_ l^−1^)	(µmol O_2_ l^−1^)	(µmol O_2_ l^−1^)	(µg C)	(µg C µg C^−1^ d^−1^)	(µg C µg C^−1^ d^−1^)	(µg C µg C^−1^ d^−1^)
**Foraminifer1**	22.66	228.51	0	52.66	0.027	0.015	0.003
**Foraminifer2**	26.43	220.22	0	56.86	0.030	0.015	0.003
**Foraminifer3**	46.44	219.37	0	55.64	0.036	0.014	0.002
**Foraminifer4**	48.27	221.45	0	49.86	0.040	0.016	0.002
**Nematode1**	105.25	229.59	0	122.50	0.045	0.012	0.001
**Nematode2**	311.28	226.79	1.21	116.50	0.049	0.008	0.001
**Nematode3**	64.71	164.82	0	120.56	0.043	0.014	0.002
**Nematode4**	259.38	195.03	16.12	111.76	0.060	0.010	0.001
**Gastropod1**	50.91	228.77	0	89.81	0.050	0.019	0.003
**Gastropod2**	57.88	226.92	0	95.62	0.035	0.013	0.002

Respiration rates are shown at 100%, 10% and 1% oxygen saturation (at 20°C and salinity 34).

The observed oxygen trajectories can be described accurately with a Monod function (see Materials & Methods) of respiration rate as a function of [O_2_] (Fig. 2A–C). The highest respiration rates in oxic conditions are observed for the Nematode and Gastropod (Table 1). When [O_2_] decreases, the respiration rate of the Nematode quickly declines (Fig. 2E), which is determined by its high half-saturation constant k_s_ (Table 1). The Foraminifer (Fig. 2G) and Gastropod (Fig. 2H) have a lower half-saturation constant k_s_ and hence a slower decline in respiration rate with decreasing [O_2_]. The Nematode is thus more sensitive to [O_2_] changes than the Foraminifer and the Gastropod.

The Ostracod treatment shows a very distinct behavior in the respiration measurements (Fig. 2D, H): The maximal respiration R_max_ 100% is similar for all Ostracod replicates. However, the Monod model does not adequately fit the respiration behavior below 85–120 µmol O_2_ l^−1^. The oxygen consumption of replicates 1 and 2 levels off (i.e. respiration becomes nearly zero) to an [O_2_] of about 100–150 µmol O_2_ l^−1^ (i.e. O_2,min_) for some hours, after which a linear decrease in [O_2_] commences. From this linear model, respiration clearly differs among replicates: while Ostracod 1, 3 and 4 return to a respiration rate that is 58–91% of R_max_ at oxic conditions, Ostracod 2 starts consuming oxygen at a rate that is even 16% higher than R_max_ at oxic conditions (Table 2, Fig. 2H).

**Table 2:** Coefficients of the non-linear (NL) and linear models (L) for the Ostracod respiration measurements.

Treatment	k_s_ NL	O_2 initial_ NL	O_2,min_ NL	Biomass	R_max__day (100%)	R_day L (<10%)
	(µmol O_2_ l^−1^)	(µmol O_2_ l^−1^)	(µmol O_2_ l^−1^)	(µg C)	(µg C µg C^−1^ d^−1^)	(µg C µg C^−1^ d^−1^)
					Non-Linear	Linear
**Ostracod1**	350.00	220.88	92.94	29.70	0.053	0.031
**Ostracod2**	350.00	214.59	15.72	29.25	0.044	0.051
**Ostracod3**	350.00	199.06	0.00	37.00	0.039	0.027
**Ostracod4**	63.60	199.81	0.00	37.00	0.034	0.031

### Respiration Strategies at Suboxic Conditions

The experimental data presented here are from single-species incubations. However, two respiration strategies emerge: (1) decreasing respiration as oxygen availability diminishes (i.e. high sensitivity to a decrease in [O_2_] in the Nematode) and (2) maintaining respiration at the onset of oxygen stress (i.e. lower sensitivity to a decrease in [O_2_] in the Foraminifer and the Gastropod). These two different strategies are striking and can be explained by habitat preferences and adaptations of the studied taxa. The Foraminifer *Ammonia spp.* is known to thrive temporally in suboxic to even anoxic sediments [31], [32] and is capable of facultative anaerobic metabolism [31], [33]. Also the Gastropod *Hydrobia ulvae* has to withstand suboxic conditions during low tide [34] and avoids desiccation periods through burial in the sediment and closure of the operculum [35]. The Nematode *Enoploides spp.* is a facultative predator and is known to migrate between surface and subsurface layers in the intertidal [36]–[38]. Apart from being very mobile, there is no evidence of this species carrying chemosymbionts or being capable of anaerobic metabolism. This species might therefore be less tolerant to suboxic conditions than the before mentioned taxa. Other nematode species than *Enoploides spp.* dwell in deeper sediment layers and it would be challenging and interesting to measure respiration rates of nematodes known to thrive mainly in hypoxic sediment areas, such as *Theristus* spp. and *Sabatieria punctata/pulchra* [36], [38], [39] or *Terschellingia* spp. [11], [40].

The sudden increase in Ostracod respiration when oxygen levels dropped below ±50% of oxygen saturation can be interpreted as a stress reaction, which, to the best of our knowledge, has not been observed before for meiofauna. Ostracods are small crustaceans that are laterally protected by valves. They do not seem to be affected by oxygen until it reaches a critical level [41]. Some species are capable of anaerobiosis during which they can try to escape the unfavorable condition and/or close their valves to avoid accumulation of sulphide [42]. Closing the valves or anaerobiosis may explain why the respiration leveled off for some time at [O_2_] far above zero. At a certain moment, the Ostracods might have initiated a stress reaction in order to escape by clapping their valves and starting to consume the remaining oxygen in the vial, which would explain the sudden linear decrease in [O_2_].

### Implications at Food Web Level

Reduced respiration at low ambient oxygen availability has three important implications for the role of meiofauna in the food web of marine sediments.

Firstly, reduced respiration at suboxic conditions indicates that the metabolism of meiofauna in these sediment areas is lower than previously thought. Hence the life span of these organisms should necessarily be longer. In addition, respiration rates are also used to estimate production of meiofauna populations [43], which is then further translated in life cycle Production/Biomass (P/B; carbon turnover) ratios. Therefore, life cycle P/B estimates may also need revision.

Although the following two implications most probably apply for meiofauna in general, they will be discussed for nematodes specifically, because of their higher sensitivity to decreasing [O_2_] (i.e. high half-saturation constant k_s_).

The second implication entails the contribution of nematodes to total carbon cycling. We compare three contrasting marine sediment types for which estimates on nematode contribution to total carbon cycling have been made. We compare nematode respiration in these sediment types under oxic conditions with nematode respiration under ambient [O_2_] to assess the impact of including ambient [O_2_] in respiration estimates. The sediment types include a coastal station (fine sandy) with low oxygen penetration depth (OPD), a coastal station (coarse sandy) with high OPD and four deep sea stations with high OPD (Table 3– for calculations, see Materials and Methods). To calculate nematode respiration under ambient [O_2_], we make the assumption that the entire nematode community respires at the rate of the Nematode *Enoploides longispiculosus* here presented (Table 1). Estimates of total nematode respiration is overestimated for all sediment types (2–7 times, Table 3) when reduced respiration under ambient [O_2_] is not taken into account.

**Table 3:** Nematode density, mean individual biomass and respiration under high and ambient oxygen concentrations in two coastal stations and four deep sea stations, with indication of the overestimation factor.

Station	Reference	Depth	Month	Year	Temperature	OPD	Nematode	Mean Individual Nematode	Respiration at	Respiration at	Overestimation
							Density	Biomass (±sd)	high [O_2_];	ambient [O_2_];	Factor
		(m)			(°C)	(mm)	Ind.10 cm^−2^	(µg C ind. ^−1^)	(mg C m^−2^d^−1^)^5^	(mg C m^−2^d^−1^)^6^	R_highO2_/R_ambientO2_
**Coastal**		10	February	2003	6	8	6648	0.62±0.97	20.99	2.86	7.3
**fine sand**	[2]	10	April	2003	9.5	6	10027	0.25±0.12	32.74	8.03	4.1
		10	October	2003	15	5	6996	0.45±0.55	33.31	14.54	2.3
**Coastal**		30	October	2003	15	20	1733	0.12±0.06	3.06	0.70	4.4
**coarse sand**	[2]										
**Deep sea**	[45]	1276	June	2007	-0.8	20	1707	0.06±0.02	1.28	0.63	2.0
**Canyon**	[14], [58]	343	May-June	1999	12.6	5	1094	0.11±0.08	3.64	0.74	4.9
		3097	May-June	1999	2.6	12	1233	0.09±0.06	1.27	0.46	2.8
		4298	May-June	1999	2.5	30	497	0.05±0.03	0.31	0.21	1.5

For coastal sediments with a low OPD, the difference between the two methods of respiration calculation is highest and may even be larger if species-specific respiration rates would be applied (the respiration rate of the active *E. longispiculosus* is likely higher than less mobile nematodes in a coastal sediment community).

For sediments with a high OPD (>20 mm) the impact will be less strong. However, many nematodes dwell deeper than the maximum OPD, resulting in low respiration at ambient [O_2_]. Therefore, respiration calculated according to fully oxygenated conditions still results in an overestimation. For deep sea sediments, the species-specific respiration rate of *E. longispiculosus* is certainly too low compared to the average respiration of small deep sea nematodes. Therefore, respiration at ambient [O_2_] might be more similar to respiration estimates at high oxygen conditions in deep sea sediments.

In all, this implies that not taking into account the decreased respiration rates at lower oxygen availability in deeper sediment layers leads to a significant (2–7 times) overestimation of the contribution of nematodes to total carbon cycling in the benthic food web.

Finally, the interpretation of carbon assimilation in nematodes as a function of their daily respiration might need a revision as well. An organism’s respiration of organic matter should be balanced by its assimilation of organic matter. Many experiments aiming at measuring assimilation provide nematodes with isotopically labeled food sources [3], [44]–[48]. However, the assimilation rates of nematodes in these experiments always prove to be negligible fractions of the daily carbon necessities [3], [44], [45] based on the respiration estimates derived from the nematode’s biomass [22], [49]–[52]. The question is whether this discrepancy between assimilation and respiration is due to the choice of food source in these experiments or rather to overestimated respiration rates. Food sources of nematodes include bacteria, ciliates, diatoms and other algae, other nematodes or oligochaetes, detritus and dissolved organic matter [53]–[55]. However, when those food sources are supplied in labeled form to nematodes, they are only traced in limited amounts which make up a few percentages of the estimated daily respiration needs. Hence, the respiration estimates might be invalid. *Enoploides sp.* assimilates about 0.000357 µg C µg C^−1^ d^−1^ [44], which corresponds to 7% of its daily respiration needs (C assimilation/respiration) at 100% oxygen saturation. However, if we assume that *E. longispiculosus* dwelling at 1–2 cm depth in the sandy intertidal has access to 10% oxygen saturation, then their respiration rate is about 25% of the rate at full oxygen saturation. In this case, carbon assimilation makes up 32% of the daily respiration needs, which, although still insufficient, represents a 4-fold increase in the contribution of this food source to the daily respiration needs.

### Conclusions

The oxygen turnover model predicts a tight coupling between ambient [O_2_] and nematode body [O_2_]. These conditions favor long and slender nematodes in sediments with a low ambient [O_2_]. The model further shows that oxygen within the nematode body is consumed in the order of seconds (even at reduced respiration rates at ambient [O_2_]), which implies that frequent migration between suboxic and oxic layers is not a viable option.

Respiration rates of all measured meiofauna organisms slowed down as a response to a decrease in ambient [O_2_]. Compared to the Nematode *E. longispiculosus*, the Foraminifer *A. beccarii* and the juvenile Gastropod *H. ulvae* had a lower metabolic sensitivity for declining [O_2_]. The Ostracod showed a behavioral stress response when ambient [O_2_] reached a critical level.

The low respiration rates at low ambient [O_2_] imply that meiofauna in suboxic sediments must have a lower metabolism, which has important repercussions for the assumed life span and P/B ratio of these deep sediment dwelling organisms. For nematode metabolism specifically, the respiration rates as a function of [O_2_] have two important implications: (1) a 2-7 -fold lower contribution of nematode respiration to carbon cycling, especially in oxygen-stressed environments and (2) although still insufficient to fully meet the daily carbon needs, a 4-fold increase in the contribution of this food source to the daily respiration needs.

## Materials and Methods

### Respiration Measurements

Respiration measurements were performed in micro respiration chambers. Detailed methodology can be found in [30], but briefly consists of the following. The micro-chambers had a very small built-in fluorescent oxygen sensor spot (Presens, Germany), where oxygen acts as a dynamic fluorescence quencher of a luminophore in a polymer matrix. An optic fibre connected to the OXY 4 oxygen meter (Presens, Germany, source and receiver of optical signal connected to a PC for continuous monitoring) was guided and positioned from the outside of the chamber on the spot. A glass vessel (1 cm high and an inner diameter of 0.9 cm with a wall thickness of 1.5 mm), equipped with a coated magnet, is filled with a layer (ca. 1 mm thick) of glass beads (100–110 µm diameter, Sartorius, Germany) which constitutes the substrate. Finally, the chamber is filled with fauna and then to the rim with seawater. This assembled chamber was then positioned with spot directly in front of the polymer optical fibre (to transmit and receive optical signals, position fixed and ensured through a plastic pipe as guide) in a water bath maintained at 20°C in a temperature-controlled laboratory maintained at the same temperature. The temperature bath was placed on a magnetic stirring table to ensure sufficient water mixing within the vessels. In total, 4 chambers were simultaneously incubated with polymer optical fibres connected to a 4-channel oxygen meter (OXY 4).

Test organisms included the Foraminifer *Ammonia beccarii*, the Nematode *Enoploides longispiculosus*, the juvenile Gastropod *Hydrobia ulvae* and an undefined Ostracoda species. These organisms were handpicked from intertidal sediment free of mud (fine particles removed through sieving) collected during low tide at different locations in the Westerschelde estuary (southwest Netherlands) (Table 4). After several cleaning steps in seawater, organisms were transferred to the micro respiration chamber. Chambers filled with only seawater of the final wash were used as a control for bacterial contamination. Per run of four chambers, two controls and two fauna incubations were conducted. The incubations were initiated with online measurements pre-set every 5 minutes until oxygen was depleted. At the end of the experiment most of the animals were found in the substrate. Only runs in which all animals were recovered alive were considered (Table 4). Animals were then stored at -20°C for size and organic carbon measurements.

**Table 4:** Number of individuals incubated in each respiration measurement with their average dimensions and biomass organic carbon (average of two replicate sets for each taxon, with standard errors in brackets), nd = no data, from [30].

Taxon	# of Ind.	Max. length	Max. width	Individual
		(µm)	(µm)	Biomass (µg C)
Ostracoda sp.	45	409 (40)	203 (12)	0.66 (0.01)
	50			0.74 (0.01)
Nematoda	50	nd	nd	2.38 (0.06)
*Enoploides longispiculosus*	44			2.63 (0.10)
Foraminifera	24	492 (90)	442 (67)	2.28 (0.09)
*Ammonia beccarii*	24			2.19 (0.12)
Gastropoda	7	1088 (64)	763 (52)	13.24 (0.42)
Juvenile *Hydrobia ulvae*				

All specimens were then transferred to silver boats, freeze dried and acidified; complete dissolution of the shells was verified microscopically. Carbon content was determined following [4], [56] on a Carlo Erba CN analyser (Table 1). Oxygen consumption (µmol O_2_ l^−1^ h^−1^) was corrected for the average value recorded for the blank incubations (10–20%). This value multiplied by the water volume (300–320 µl) and divided by the number of specimens incubated. This was converted to respiration rates per unit biomass using absolute organic carbon content measured as described above.

### Respiration Model

The changes of [O_2_] (µmol O_2_ l^−1^) in time in the incubation chambers were reproduced with the following differential equation:
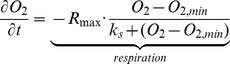
In which the decrease in [O_2_] is fitted with a Monod equation, in which *R_max_* is the respiration at the start of the measurement, *k_s_*is the half-saturation constant at which respiration is 50% of *R_max_*. The *k_s_*also signifies how fast the respiration rate slows down with depleting [O_2_] (i.e. metabolic sensitivity to a decrease in [O_2_]). The initial [O_2_] in each chamber was used to initialize the model. The parameter *O_2,min_*is given a value only in the Ostracod replicates 1 and 2 and Nematode replicates 2 and 4 to allow reproducing this specific respiration pattern: after an initial Monod-type decrease with declining [O_2_], the time course clearly starts deviating. The parameter *O_2,min_*has the interpretation of the [O_2_] at which the organism dies (i.e. no more oxygen utilization) if the Monod-type respiration would continue. In the other treatments, [O_2_] reaches zero and therefore *O_2,min_*is zero.

The equation was fitted using the non-linear fitting routine provided in the R package FME [57]. Initial [O_2_] for each incubation was set to the [O_2_] readings from each chamber. Initial parameter values for the fitting procedure for R_max_ and *O_2,min_*were taken from a linear fit of the data, whereas the initial value for the fitting of k_s_ was 10.

The linear last part of curve in the Ostracod treatments was fitted with a separate linear part where the respiration is simply R_max_, without a rate-limiting term.

The fitted respiration rates (µmol O_2_ l^−1^ h^−1^) were further converted to daily respiration in terms of carbon (Resp_day, µg C µg C^−1^ d^−1^), assuming that 1 mole oxygen is consumed to respire 1 mole carbon:
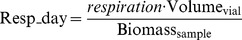



In a next step, the daily respiration in terms of carbon (Resp_day) was fit as a function of the available [O_2_] at 100%, 10% and 1% oxygen saturation (at 20°C and salinity 34) following:
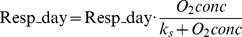



### Calculation of Nematode Contribution Total Carbon Cycling

Individual nematode biomass was calculated for the coastal sand and canyon data set [2], [14] by allometric conversion of nematode Length and Width data to wet weight [49]:



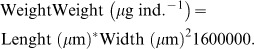



Then, these wet weights were further to dry weights [50]:
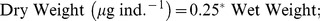
and weight in terms of carbon [51]:



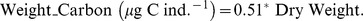



For the deep sea data set [45], Weight_Carbon (µg C ind. ^−1^) was directly available.

Respiration at high [O_2_] is the sum of individual weight-dependent respiration rates [52]:
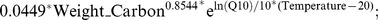
with *Q*10 assumed 2 and Temperature from Table 3.

Respiration at ambient [O_2_] is the sum of individual respiration in each sediment depth layer:
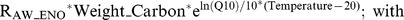


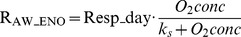
and Resp_day and k_s_ respectively the biomass-specific respiration rate and half-saturation constant of Enoploides. O_2_conc is the ambient [O_2_] (AW), inferred from the shape of the oxygen profile and/or the OPD mentioned for each station in Table 3.

As such, R_AW_ENO_ can be R_max__day (100%) in the first fully oxygenated cm of the sediment, declining to R_max__day (10%) in the suboxic parts of the sediment and eventually to R_max__day (1%) in the sediment layers below the maximum OPD.
